# A Privacy Preserving Scheme for Nearest Neighbor Query

**DOI:** 10.3390/s18082440

**Published:** 2018-07-27

**Authors:** Yuhang Wang, Zhihong Tian, Hongli Zhang, Shen Su, Wei Shi

**Affiliations:** 1 Research Center of Computer Network and Information Security Technology, Harbin Institute of Technology, Harbin 150001, China; apple125110@gmail.com (Y.W.); zhanghongli@hit.edu.cn (H.Z.); 2 Cyberspace Institute of Advanced Technology, Guangzhou University, Guangzhou 510006, China; johnsuhit@gmail.com; 3 School of Information Technology, Carleton University, Ottawa, ON K1S 5B6, Canada; WeiShi@CUNET.CARLETON.CA

**Keywords:** location privacy, nearest neighbor query, noise addition, R-tree, wireless sensor localization, Internet of Things

## Abstract

In recent years, location privacy concerns that arise when using the nearest neighbor query services have gained increasing attention, as such services have become pervasive in mobile social networks devices and the IoT environments. State-of-the-art privacy preservation schemes focus on the obfuscation of the location information, which has suffered from various privacy attacks and the tradeoff of the quality of service. By noticing the fact that the user’s location could be replaced by their surrounding wireless sensor infrastructures in proximity, in this paper, we propose a wireless sensor access point-based scheme for the nearest neighbor query, without using the location of the user. Then, a noise-addition-based method that preserves user’s location privacy was proposed. To further strengthen the adaptability of the approach to real-world environments, several performance-enhancing methods are introduced, including an R-tree-based Noise-Data Retrieval Algorithm (RNR), and a nearest neighbor query method based on our research. Both performance and security evaluations are conducted to validate our approach. The results show the effectiveness and the practicality of our work.

## 1. Introduction

Geolocation information of both people and devices is becoming increasingly significant. On one hand, people can use their locations to receive various services (such as the nearest neighbor query in mobile social networks) that enhance their daily activities. On the other hand, advancements in the wireless sensor localization have enabled the development of applications to obtain Points of Interest (POI) with the smartphone; thus, people can acquire the location of nearly anything by allowing the devices to perceive its surrounding wireless sensors and hence to know their current geolocations, and various applications will be available, such like the IoT performance appraisal [1] and the mobile entertainment [2].

**Figure 1:**
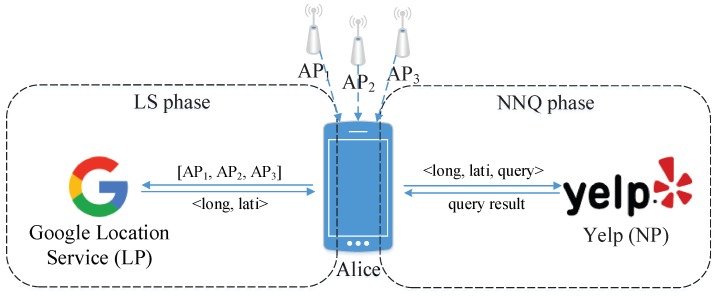
An example of LS and NNQ.

In the view of the positioning procedure, the third-party geolocation services have been adopted in most real world scenarios in which location is required. A geolocation service (LS) is a centralized third-party information service through which users retrieve their geolocations by sending information concerning nearby wireless sensor to the geolocation service provider (LP) such as Google, Skyhook, Baidu and so on. Compared to some other wireless sensor localization technologies (such like the RFID identification method proposed in [3]), the more ubiquitous of these services are the Wi-Fi-based fingerprint localization services, and this kind of service can be called as the basic level fuction in nearly every smartphone. The providers of such LSs are often gigantic companies such as Google and Apple. On the other hand, the nearest neighbor query services (NNQ) are offered from the other side of the phase, a NNQ provider (NP) receives user’s location and then performs the NNQ services. Figure 1 shows an example of these two phases, when Alice needs to know the nearest restaurant in proximity, she first requests her geolocation from the Google location service (Google acts as the LP) during the LS phase, then during the NNQ phase, she sends the geolocation and the query criteria to Yelp (Yelp acts as the NP) to exchange the information she needs. In such a process, both LP and NP will know Alice’s geolocation, obviously, as does any third-party information service, these LSs and NNQs raise privacy concerns due to their very nature. This privacy issue is serious and needs to be solved.

Unfortunately, the efforts prior studies have devoted to preserving location privacy in NNQ phase are unsuitable for solving the location privacy problem in LS phase because the protected information involved in the two phases is totally different. Consider the approaches that add noise as an example; in which at the most basic level, users can mix some completely random “dummy” coordinates with their real locations. Researchers have tried every means possible to improve the obfuscation (as well as other privacy aspects) of the dummy locations. However, in an LS phase, the noise data must instead consist of signal space vectors, which demands stricter patterns than that of geolocation-based approaches.

Many works have been devoted to preserving location privacy in the NNQ phase. In contrast, few studies have focused on the LS phase. This indicates that the location privacy must be protected by two different schemes that work on both the LS and NNQ phase independently, this situation goes against the entire issue of location privacy, especially when the LP and NP are the same entity, the efforts on both sides will be in vain.

In this paper, we proposed a wireless access point based scheme to preserve location privacy in NNQ services. We formally modelized the spatial features of an access point (AP) and demonstrated what types of APs can be used as noise data. Based on this model, we used the combination of APs surrounding the users directly as the query criteria, no location information is needed in our approach, and this greatly strengthen the location privacy.

The major contributions of this paper are summarized as follows:

1. From a theoretical point of view, we proposed a formal model for the spatial distribution of APs that can be used to verify whether a set of APs qualifies for use as noise data in LS phase.
2. We proposed an R-tree-based Noise Data Retrieving Algorithm (RNR) to generate the noise data set for a specified real location. RNR generates a high-quality noise data set and simultaneously optimizes the performance, making our approach suitable for mobile and IoT devices.
3. We proposed a NNQ method based on the APs information, NP can use the APs as the query criteria directly to search the nearest neighbor.
4. A comprehensive evaluation of both, solution performance and security aspects, show the effectiveness and the practicality of our work.

The rest of this paper is organized as follows. Section 2 briefly reviews the related work, and Section 3 outlines the model used to determine the noise data. We describe the design details of our approach in Section 4. Section 5 and Section 6 provide theoretical analyses and experimental evaluations, respectively. Finally, Section 7 concludes the paper and suggests future work.

## 2. Related Works

Much attention to location privacy has been paid to the phase of NNQ over the past decade. The approaches are comprehensively surveyed in [4,5,6]. The threat models of these approaches are similar: they prefer to treat the Location Based Service (LBS) providers as untrusted adversaries with extensive background knowledge. Based on this framework, their primary purpose is to prevent the LBS providers from knowing the users’ accurate locations while at the same time retaining as much of the LBS functionality and service quality as possible. Researches based on obstruction [7,8,9], clique region [10], encryption [11] and obfuscation [12] are widely adopted for the detail of their implementations.

Few efforts have been made to preserve privacy from the other side of this problem: the location privacy in LS phase, which is equally important. Reference [13] clearly described this privacy issue in the third party wireless sensor localization service, but offered no computational solution, and reference [14] introduced a detection method of privacy attack against the NNQ. In [15], the authors described a location privacy threat called a location spoofing attack, in which the attackers counterfeit a smartphone’s original wireless sensor information and imitate the real user to request the user’s location from the LP. Then, the method proposes a reliability determination algorithm to cope with this threat. However, their threat model regarded the LP as trusted, which is unrealistic in the real world. The encryption-based approach was proposed in [16,17] to cope with privacy threats in Wi-Fi-based LSs. However, the encryption procedure is considered to be time-consuming for mobile devices; moreover, it is designed for Wi-Fi-based localization only, which limits its widespread adoption.

Finally, from the view of the system architecture model, the trusted third party (TTP) broker is widely adopted in some literature. TTP is needed in some location privacy researches often because their preservation scheme needs the information that beyond the user’s ability, or the algorithm is too time-consuming for the normal devices. However, the TTP broker is considered to be lack of practicality, since it is unrealistic in the real world, the absolutely trusted role is hard to define in the real world threat model.

## 3. Preliminaries

In this section, we first describe the wireless localization technologies in brief, then we discuss the spatial distribution features of APs and introduce the graph structure to model it. Finally, we present the system model our system adopts.

### 3.1. Wireless Localization Technology

Wireless localization is widely used in mobile environments and IoT scenarios, and fingerprint localization is one of these technologies, it was first designed as an indoor localization scheme but was subsequently shown to perform reasonably well in outdoor environments. In both application scenarios, the underlying techniques tend to be the same. Generally, fingerprint localization approaches transform signal space vectors into Euclidean space vectors by performing a two-step procedure. The offline step is conducted by the LP and generates a localization database that includes the signal space beacons (commonly termed “fingerprint”) in specified areas. Depending on the technological details, this database may contain either the geolocation of each AP or only the fingerprint database. During the online step, the LP estimates a device’s geolocation by searching the database using fingerprint vectors acquired from a user’s device. In the current environment, the APs massively exist in the city areas.

Besides fingerprint technology, the triangulation localization technologies are also proposed. The basic principle of triangulation method is: If the geographical coordinates of three reference wireless nodes are known, the location of the user can be calculated by using either the distance or the direction by performing the trigonometric calculation. Based on the geometric properties of triangles, different methods can be used to calculate the location, for example, the angle of arrival (AoA) and the time of arrival (ToA).

Although the state-of-the-art of localization technologies use different estimation methods, these differences have little effect on the user side, which is convenient for designing a universal location privacy preserving algorithm in LS phase.

### 3.2. Spatial Distribution Model of AP

The basic principle in state-of-the-art wireless localization technologies requires the device to sense signal space features in its proximity, collect them, and send this so-called “fingerprint” information to the LP through the network. Then, the LP estimates a specific geolocation associated with the received fingerprint and sends it back to the device. From the device viewpoint, the details of the type of localization technology (for example, AoA, ToA and fingerprint) the LP is using are irrelevant; the device always performs almost the same tasks, which are, sense and record the information of its surrounding APs and then send them to the LP.

**Figure 2:**
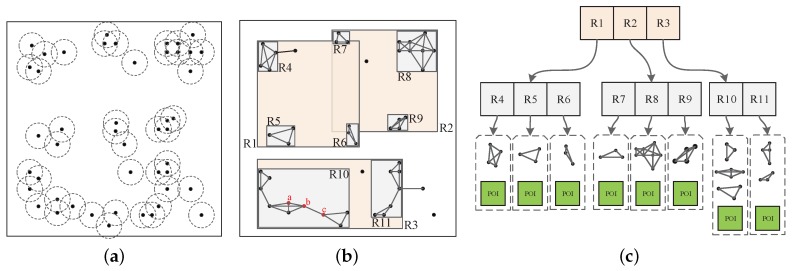
The basic idea of our approach. (**a**) AP spatial distribution and their signal coverage area; (**b**) undigraph *G*; (**c**) R-tree *T* of the clique snippets.

The key requirement of noise addition is that the spatial distribution of a noise data set should accurately mimic the real world fingerprint which is sensed by the device. In this paper, we use an undigraph to model the spatial distribution of APs. As shown in Figure 2a,b, if we define a vertex as an AP and the edge between two vertexes as the “overlapping” relation of each AP’s signal cover area, then a “fingerprint” cluster-like feature can be modeled as a complete subgraph of the entire graph.

Formally, given the real “fingerprint” data set *F* in the undigraph G(V,E), which v∈V represents the AP, and e∈E represents the overlapping relation between two vertexes, we wish to find noise data that is similar to the real data set, that is, to find a series of vertexes $V_{noise}$:

$$\left[\begin{array}{ccc} v_{11} & \cdots & v_{1n}\\ \vdots & \ddots & \vdots \\ v_{k1} & \cdots & v_{kn} \end{array}\right]$$

in which

1. $\left\{v_{i1},\cdots ,v_{in}\right\}$ (along with the edges between them) consists a complete subgraph in *G*,
2. $V_{noise}\cap F=\emptyset$, and
3. n=|F|.

The second limitation is not a necessary condition for the noise data set, it is defined mainly for the privacy concerns. Utilizing this model, the noise generation problem in LS phase can be transformed into a clique-finding problem in an undigraph, and we can use the clique of APs directly to represent the location information of a user, an then use it directly to perform the NNQ.

It is important to note that the brute-force algorithm for finding a clique in an undigraph requires exponential time [18]; thus, this noise generation method limits the utilization potential due to device limitations (processing power, delay and energy constraints), especially in mobile and IoT environments. To address this problem, we design the RNR algorithm, which is discussed in Section 4.2.2.

### 3.3. System Model

In this study, we consider the NP as an honest-but-curious attacker who provides NNQ honestly but will infer and collect client locations. The NP knows all the NNQ requests as well as the details of the privacy preserving technology used by the devices. Beyond that, we consider both the device and the network to be trusted because other security issues are out of the scope of our discussion, and could be satisfied by utilizing the corresponding privacy preservation schemes such like [19,20].

The graph model of APs (denoted as *G*) of the whole area is possessed by the NQ, and the user holds a partial of the *G*, he will sense and collect the historical AP data and use them as the candidate of noise generation. We use the direct communication model to perform our approach and no third party broker are needed to perform the preservation for the user in our scheme.

## 4. Our Proposed Schemes

To address the privacy issue and the implementation quandaries described above, in this paper, we first propose a noise addition approach that can preserve the location privacy of users from third-party geolocation services. Then, the AP-based NNQ query method will be introduced. In this section, we detail the design of our work.

### 4.1. System Overview

Our approach is based on the fundamentals of the localization technologies; therefore, we do not need to make any assumptions concerning infrastructure or application scenarios to apply our system. Additionally, our approach requires no cooperation from the LP, which is important in practical privacy preservation schemes.

**Figure 3:**
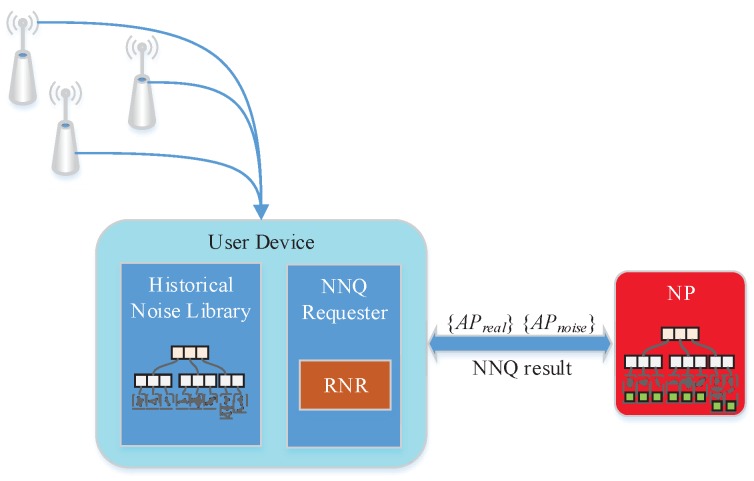
System architecture.

Figure 3 shows an overview of the system architecture, which contains two main modules: a noise library containing the identifying information of APs and their spatial distribution topology model. In our work, the noise library is owned by the user. The NNQ requester module first utilizes real data to request the noise data set from the noise library; then, it requests an NNQ from the NP using all these “fingerprints”. Finally it selects the real query result and sends that to higher-level applications.

To describe our approach, we first introduce our noise-screening strategy and the RNR algorithm. Then, we describe the AP-based NNQ service.

### 4.2. Noise Generation Scheme

We first introduce the modified noise data retrieval method which can greatly shorten the preservation time. Then, on the basis of the proposed method, we further take the privacy concerns into consideration. Finally, we introduce our noise generation scheme.

#### 4.2.1. Modified Noise Data Retrieval

As illustrated in the preliminaries, we model the APs spatial topology as the undigraph and transform the noise data set retrieval into the clique search problem. However, this approach is too time consuming when the undigraph is large. To address this problem, we first locate and mark the “dense areas” of the undigraph; then, we cut the undigraph into snippets.

Given the undigraph *G* which is defined in Section 3.2, We use the clustering coefficient of each vertex $v_{i}$

$$c_{i}=\frac{|\{e_{jk}:v_{j},v_{k}\in N_{i},e_{jk}\in E\}|}{k_{i}\left(k_{i}-1\right)}$$

to represent how likely this vertex and all its neighbor are to compose a complete subgraph, here $e_{jk}$ denotes the edge between the vertex $v_{j}$ and $v_{k}$, $k_{i}$ is the number of the neighbours of $v_{i}$, and $N_{i}$ is the neighbours of $v_{i}$. According to the definition, this coefficient of the three red vertexes a, b and c in Figure 2b should be 1, 0.58 and 0.67, respectively. In particular, we can assert that a complete subgraph exists when this coefficient is 1. Given the global undigraph of APs (denoted as *G*), we first define the clustering coefficient *c* as the weight of each vertex. Then, using *G* and the threshold *c*, we divide *G* into snippets in which each snippet is a clique with several vertexes. The value of *c* can be adjusted to control the noise library’s size; when *c* is 1, all the noise sets in the noise library will be perfect candidates for our approach.

When the number of snippets is large, to further improve the retrieval of the noise data set, we use an R-tree-based data structure to construct the noise data set and facilitate retrieval. As Figure 2c shows, the R-tree *T* was built over the entire area to store the AP snippets. In our work, we actually focus on the distribution feature of the APs, it is not necessary to store every AP geolocation; consequently, the leaf nodes in each snippet contain only one geolocation for each snippet, which represents one vertex of that snippet.

Based on the constructed snippets of *T*, our approach can retrieve a noise data set that is both localizable and efficient. Next, we discuss further privacy issues involving noise data.

#### 4.2.2. Enhancing Privacy in Query Probability

In our work, we choose those noise data sets that can induce NP to generate “dummy” locations for the device. Moreover, to further enhance the privacy level of our approach, we consider the situation that the NP may perform the collusion attack with LP, and they could locate user by the positioning technologies. To conquer this threat, the dummy location should have a “query probability” sufficiently similar to the real location. In a grid-divided area, the query probability (denoted as *q*) of each grid cell is the probability of that cell being queried, which is proportional to the number of times the location was queried in the past:

$$q_{ij}=\frac{|LS_{ij}|}{|LS_{global}|}$$

**Figure 4:**
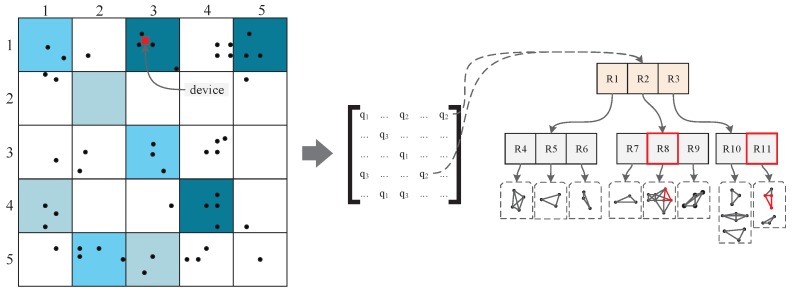
Noise data set concerning the query probability.

Figure 4 shows the characteristics that our noise data should satisfy. In the figure, similar cell color depths represent similar *q* values. Here, device in cell $c_{13}$ should choose a noise data set that can induce LP from localizing into cells $c_{15}$ and $c_{44}$. In our work, benefitting from the R-tree snippet noise library, we can achieve noise data set optimization easily by simply using the grid cell area as the query criteria to the R-tree snippet library. Based on the q-matrix (denoted as $M_{q}$), we select those cells whose query probability is similar to $q_{real}$ when searching the snippets in *T*. As a result, we obtain a noise data set that will localize a geolocation similar to the real location (i.e., representing a higher privacy level).

Combining the above-mentioned designs, the design of the RNR algorithm is shown in Algorithm 1. The RNR algorithm takes the $M_{q}$ and the query probability of the real data set as the input and retrieves *k* noise data sets as the output. In detail, we use a random method to filter out surplus noise candidates that satisfy our need, which includes selecting random *k* cells among all the candidate cells with similar *q* values, and randomly choosing vertexes in a snippet when the number of vertexes in this snippet is greater than the number of vertexes in the real data set. In contrast, when fewer than *k* noise data sets satisfy the conditions, the algorithm iterates over the process, adjusting the *q* value in each iteration to extend the search scope.

**Algorithm 1** RNR algorithm**Require:**  $M_{q}$;  $q_{real}$; **Ensure:**  *k* noise data sets $N_{1},\cdots ,N_{k}$;1:SNIPPETS = searchTree($q_{real}$);2:**if**sizeof(SNIPPETS)≥k**then**3: **for** each snippet in SNIPPETS **do**4:  **if** vertex number of $snippet_{i}$ is larger than vertex number of real data **then**5:   $NoiseSet_{i}=\text{random\_vertex}\left(snippet_{i}\right)$;6:  **end if**7: **end for**8: randomly choose $N_{1},\cdots ,N_{k}$ from NioseSet;9: **return** $N_{1},\cdots ,N_{k}$;10:**else**11: reduce *q* and repeat from line 1;12:**end if**


#### 4.2.3. The AP-Based NNQ

As *G* contains only the distribution of APs, it cannot be used directly to perform the NNQ. In our work, we further enhanced *G* by enable it to estimate the distance between each vertex. To realize this front, we estimate the distance between two overlapped APs by performing the signal strength distance conversion. In this paper, the pervasive function of this conversion was adopted, which can be formally described as:

$$d=10^{\frac{\left|rssi\right|-A}{10\times n}}$$

Here rssi is the received signal strength of AP in some location of the overlap area, *A* is absolute value of signal strength at one meter distance to the source sender and *n* indicates the path loss attenuation factor. Both of these parameters in this equation can be obtained by measuring (e.g., the rssi) or inherit (e.g., the *A* and *n*) from the background knowledge. Then, the estimation of the distance between APs (denoted as $d_{AP}$) can be calculated out by the trigonometric function calculation. We further use this distance as the weight of edges of *G*, the NP can use *G* to estimate the distance between the users and all its neighbors. The specific procedure of the AP-based NNQ is as follow:

step 1: Select the vertex with the largest *c* in the query criteria as the anchor AP, denoted as $v_{a}$.

step 2: Choose those vertexes from the neighbor of $v_{a}$ in *G* which are combined with the POIs, denoted as $v_{c}$, if no vertex in neighbor of $v_{a}$ is combined, repeat this step to all the neighbors of $v_{a}$’s neighborhood.

step 3: Calculate the estimated distance between $v_{a}$ and each vertex in $v_{c}$ and return the POI of the nearest vertex.

In our work, we use this AP-based NNQ method to replace the traditional NNQ. Benefit from this method, NNQ could be carried out without the use of location of user, hence, it enhances the location privacy to a large extend. Further evaluation is shown in the following section to verify this improvement.

## 5. Analysis

In this section, we discuss the performance and the security aspects of our work. For the ease of presentation, we list in Table 1 the symbols and abbreviations used in this paper.

### 5.1. Performance

As our method is carried out on the mobile device, we mainly considered two types of important system overhead of a mobile device: the time complexity and the memory overhead.

#### 5.1.1. Time Complexity

We focus on the time complexity of RNR algorithm mainly because it leads to runtime delays in the LS. The RNR involves two computing phases. The first phase is grid cell selection from the $M_{q}$. Here, we choose *k* cells, which form the search condition of the second phase. The time complexity of this operation is $O(n^{2})$. The second phase searches snippets in the R-tree. The average theoretical time complexity of this operation is $O(\log^{M})$, where *M* denotes the number of snippets contained in *T*. Therefore, the time complexity of RNR can be described as $O\left(n^{2}\right)+O\left(\log^{M}\right)$, which is considerably smaller than the time complexity of the brute-force clique discovery algorithm.

#### 5.1.2. Memory Overhead of Device

The memory overhead of our approach depends primarily on the size of AP identifiers in the real world. Generally, the overall storage cost (*M*) can be calculated as:

M=(sizeofAPidentifier)×(numberofAPs)

In current realistic situations, the most common identifiers LPs used to identify APs are MAC addresses (and sometimes, IPv6 addresses in an IoT environment). Both MAC addresses (6 byte) and IPv6 addresses (16 byte) are small relative to current device memory capacity. Therefore, our approach involves only a small memory overhead for the device. This situation will be shown more clearly in the evaluation section.

### 5.2. Deviation

Our method uses the APs as the query criteria of NNQ instead of the location, as the result, a certain degree of deviation could occur which is caused by the estimation error. However, the average deviation of the state-of-the-art estimation scheme are 20–50 m in the urban area, such magnitude of deviation is almost as fair as the estimation deviation of the positioning technologies. Therefore, it could be considered as equivalent to use the AP-based method and the location-based method.

### 5.3. Privacy

In Section 3.3, we briefly introduced the threat model used in our approach. In this subsection, we first introduce the privacy attacks we are concerned with in this paper; then, we show a security analysis in terms of each attack type.

#### 5.3.1. Direct Observation

In this attack, the NP obtains a device’s location during the NNQ operation. In our approach, we use the APs as the query criteria of NNQ, no location information is needed in the entire process. In other words, theoretically, NP cannot know any information about the user’s location, and the location privacy of the user is under an absolute preservation.

#### 5.3.2. Statistical Inference Attack

In more complex situations, since the collusion attack could be occurred, and the LP knows the global query possibilities, they may use APs to locate the user’s location and even perform the statistical inference to narrow the guess range within the set *C*. In the most fundamental inference attack with which we are concerned, the LP will always assume that the location with the highest *q* is the real location.

Because the *k* value cannot depict the success rate appropriately under this type of attack, we instead use the entropy of *C* as the metric to measure the probability that the LP will successfully locate the device. Here, we first define $p_{i}$ as the probability of the $i^{th}$ geolocation in *C* as the real location, apparently $p_{i}=\frac{q_{i}}{\sum_{i=1}^{\left|C\right|}q_{i}}$. Then, the entropy can be represented as

$$H=-\sum_{i=1}^{\left|C\right|}p_{i}*\log_{2}p_{i}$$

Based on the definition of *H*, a larger *H* represents a higher privacy level.

**Theorem** **1.** *The RNR is resistant to statistical inference attack.*

**Proof.**  From Algorithm 1, because the RNR uses only grid cells with *q* values similar to the $L_{real}$ as the query criteria, the resulting

$$\left\{q_{1},\cdots ,q_{k}\right\}$$

will all be similar to the $q_{real}$. This causes *H* to be very close to 1; thus, the LP can make only a wild guess (falling back to the direct observation attack) which has low reliability.

On the other hand, if the noise library cannot supply a sufficiently high-quality noise data set (i.e., the RNR returns fewer snippets than needed), *H* increases, and the privacy level declines. Nevertheless, in such cases, the RNR can still resist statistical inference attacks due to the long randomization period. The adoption of the randomization approach causes side-effects to the LP’s knowledge of the global query possibilities: after a sufficiently long period of system implementation, the query probability of each cell approaches equality, and the average *H* will be approximately 1. This causes LP to lose the advantage of global query probability when performing such an attack. ☐

## 6. Experimental Results

In this section, we evaluated the performance of our approach through several experiments corresponding to overhead and privacy. We used the Wi-Fi-based Google Localization Service to represent LP, and we implemented our privacy preservation method (including the R-tree maintenance, noise generation) on an Android 7.0 OS mobile device with a Qualcomm Snapdragon 821 2.15 GHz CPU and 4 GB of RAM. To use the noise data set when requesting the LS, we overwrote the relevant system code, allowing us to send custom information to the LP. In our experiments, from a privacy preservation viewpoint, we do not need to consider how Google performs its localization. We simulated the NP with the ability of performing the AP-based NNQ procedure on our Lenovo PC.

We used an open Wi-Fi dataset that includes 123,593 Wi-Fi hot spots in Tokyo, Japan along with their MAC addresses and geolocations. To simplify the spatial distribution of these APs, we transformed the geometric coordinates (latitude and longitude) into two-dimensional Euclidean space coordinates through Gauss-Kruger projection.

### 6.1. Performance

We evaluated the system overhead of our approach from two aspects: CPU time and the size of the noise library.

#### 6.1.1. CPU Time

**Figure 5:**
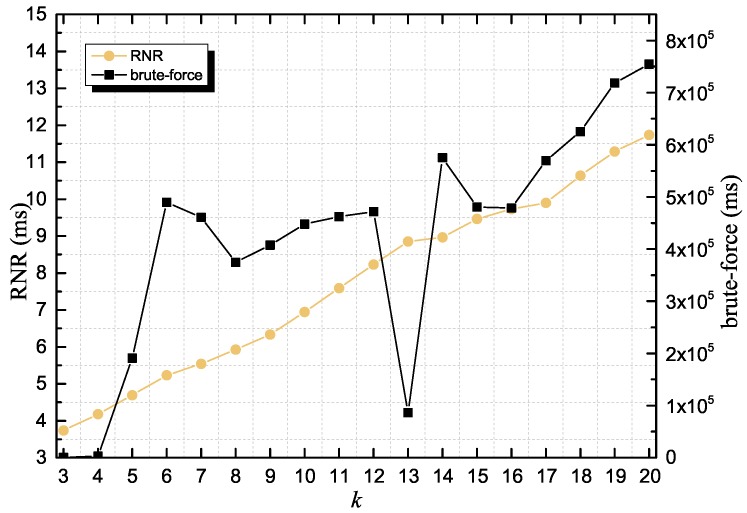
CPU time of RNR and the brute-force algorithm as *k* increases from 3 to 20 using a fixed δ of 5.

When acquiring the CPU time, we focus our attention on RNR due to its runtime delay to NNQ. We compared RNR to the brute-force clique discovery algorithm. Figure 5 shows the effects of *k* on the CPU time of each algorithm as we executed the algorithms on their corresponding global-sized data structures (the R-tree *T* and the undigraph *G*) and increased *k* from 3 to 20. The brute-force algorithm takes considerably longer and its CPU time is highly unstable. In contrast, the RNR CPU time is lower by more than an order of magnitude than that of the brute-force algorithm and the RNR CPU time increases linearly with *k*.

#### 6.1.2. Deviation

To investigate the deviation of our scheme, we compared the AP-based NNQ result to the location-based NNQ result in the same circumstance. By repeating 50 times the simulation on the two methods in the same geolocation, we counted the total number of times when different NNQ results happen. After repeating this simulation in 240 different geolocations, the result shows that only 4.54% out of the total on average did not be the same as the location-based NNQ result, this is a fairly small and acceptable proportion compared to the total number.

### 6.2. Privacy

Considering the collusion attacks, we evaluated how much privacy our approach provides by performing the privacy attacks described earlier in this paper. The evaluations are carried out on both a global noise library denoted as *T* and several smaller noise libraries constructed by user at incremental scales ($T_{1}$, $T_{2}$ and $T_{3}$ with 10 m${}^{2}$, 20 m${}^{2}$ and 40 m${}^{2}$ respectively). For the input data of the noise generation scheme introduced in Algorithm 1, both the situations that the real location is in the cell with high and low query probability are been considered.

#### 6.2.1. Resistance on the Direct Observation Attack

**Figure 6:**
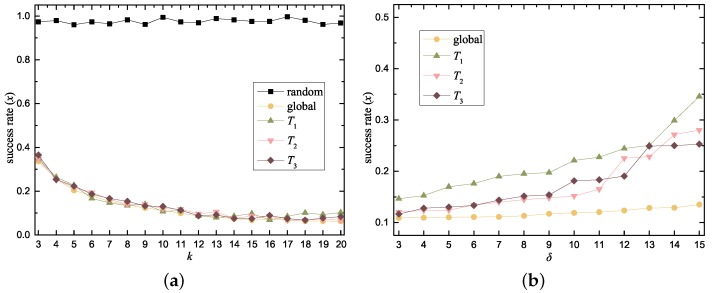
Experiment results of resistance to direct observation attack. (**a**) *x* vs. *k* with a fixed δ of 5; (**b**) *x* vs. δ with a fixed *k* of 10.

We simulated the fundamentals of this attack in which the LP performs a wild guess concerning the contents of *C*. Theoretically, the success rate of identifying the real location is $\frac{1}{k}$. Figure 6 shows the success rate (denoted as *x*) of this attack working on our method and the influences of each parameter on *x*, the success of the attack means that the attacker successfully recognized the real data out from the noise data.

In Figure 6a, we increased the number of the noise data set (denoted as *k*) from 3 to 20 and visualized the effect of *k* on the success rate *x*. We also compared our work to a no-strategy method, in which we added some (as much as the RNR output) purely random noise data into the real data. As shown in Figure 6a, the no-strategy method provides almost no privacy under this type of attack, because the LP cannot perform normal LS based on the random noise data set. In contrast, the result of RNR is consistent with the theoretical analysis. The experiment was independently repeated 500 times for each *x*, and the average *x* value decreases from 0.3369 to 0.0624 as *k* increases from 3 to 20.

Notably, the results for the self-constructed noise libraries ($T_{1}$, $T_{2}$ and $T_{3}$) are almost as good as the result using the global-library control group. This result verifies a crucial aspect of our approach: using the R-Tree construction algorithm to enable a device to conduct privacy preservation by itself.

We studied the effect of δ on *x*, where δ indicates the number of APs the LP requires for his service. As shown in Figure 6b, *x* increases slowly as δ enlarges from 3 to 15. Because a higher δ denotes more strict query criteria of the snippets in RNR, it will sometimes return a “wrong” noise data set which fails to induce the LP to return a location. This increment of *x* is more pronounced for $T_{1}$, $T_{2}$ and $T_{3}$ than for the global control group, because the smaller noise library is less diversified than the global library. Overall, the increment was less than 0.2 even in the worst case.

#### 6.2.2. Resistance on the Statistical Inference Attack

**Figure 7:**
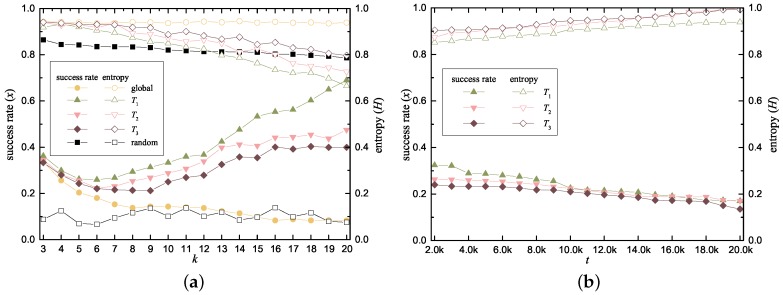
Experiment results of resistance on the statistical inference attack. (**a**) *x* and *H* vs. *k* with the initial *M*; (**b**) *x* and *H* vs. *t* with the dynamic *M*.

In this experiment, the basic statistical inference attack is carried out on privacy, which means that the LP always assumes the location with the highest *q* in *C* is the real location. We divided the global area into a grid in which each cell represents 1.2 square kilometers. Without loss of authority, the initial $M_{q}$ for this area was obtained from the Google Map API, and we simulated the LS requests with frequent patterns according to the query probability matrix. Figure 7 shows the success rate of this attack (left side scale), and the corresponding entropy of *C* that our method offered (right side scale).

Figure 7a shows the effect of *k* on the success rate *x* (solid) of this attack. We compared our work with a noise retrieval scheme that does not consider the query probability; it always uses a random grid cell as the query criteria for the noise library. Benefitting from the RNR, the entropy *H* of *C* remains satisfactorily high (approximate to 1) in the global case; correspondingly, *x* remains at quite a low value. *x* (and *H*) are theoretically independent from *k* ,which is demonstrated well in the global case; however, *k* may influence *x* from another perspective when there are insufficient cells in the query probability matrix to satisfy the similarity. In this case, RNR relaxes the similarity threshold to obtain the required number of noise data sets, which leads to the reduction of the *H* of *C* and an increase in *x*. This influence is more apparent when using $T_{1}$, $T_{2}$ and $T_{3}$ in our experiment.

We further considered the real-world situation of the dynamic matrix owned by the LP. Based on the initial query probability, the attacker must update $M_{q}$ with the location of the subsequent LS request. We studied the effect of the total number of RNR executions on *H* and *x* under the statistical inference attack. Because the noise data set continuously alters the $M_{q}$, it can be observed from Figure 7b that the *x* for $T_{1}$, $T_{2}$ and $T_{3}$ gradually declines as *t* rises from $2\times 10^{3}$ to $2\times 10^{4}$. This result provides strong evidence of the practicality of the our scheme; thus, we can claim that our approach defends against statistical inference attack efficiently in both scenarios.

## 7. Conclusions

In this paper, we investigated a pervasively adopted wireless sensor localization-based NNQ scheme, which can perform NNQ without the using of location. The noise addition-based location privacy preservation scheme is then introduced. We presented a formal model to outline the fundamental effects of adding noise in general localization techniques. Based on this model, we proposed an approach that allows the device to self-generate noise datasets that are highly confusing to the LP, this approach also enables the device to use the direct communication architecture, which is more practical for the real world implement than the TTP architecture. Despite the collusion attack, in the basic threat model, our scheme can ensure the absolute of privacy since no location is ever mentioned in our AP-based NNQ procedure. Then, we further took the collusion attack from NP and LP into consideration, and the noise addition method further ensures the high privacy level. We implemented the privacy preservation method on the mobile device, and both the performance and the privacy evaluations were performed. The results of the experiments demonstrate the effectiveness and practicality of our approach.

## Figures and Tables

**Table 1:** Glossary of Symbols and Abbreviations.

Symbols and Abbreviations	Full Term
*c*	Clustering coefficient of a vertex in *G*
*G*	Undigraph model of APs
*T*	R-tree of the AP snippets
*q*	Query probability of the grid cell
$M_{q}$	The Matrix of query probabilities of the grid cells
$C\{D_{1},\cdots ,D_{k},L_{real}\}$	Geolocation set that contains *k* dummies and 1 real location
$Q\{q_{1},\cdots ,q_{k},q_{real}\}$	Query probabilities of each cell that covers the geolocation in *C*
