# Erratum: Wang, Y. et al., A Privacy Preserving Scheme for Nearest Neighbor Query. *Sensors* 2018, *18*, 2440

**DOI:** 10.3390/s19143187

**Published:** 2019-07-19

**Authors:** Yuhang Wang, Zhihong Tian, Hongli Zhang, Shen Su, Wei Shi

**Affiliations:** 1 Research Center of Computer Network and Information Security Technology, Harbin Institute of Technology, Harbin 150001, China; 2 Cyberspace Institute of Advanced Technology, Guangzhou University, Guangzhou 510006, China; 3 School of Information Technology, Carleton University, Ottawa, ON K1S 5B6, Canada

The authors wish to make the following erratum to this paper [1]:

One the basis of the current author list, the affiliation of author Zhihong Tian should be with both 1 and 2, in accordance with the following format:

Zhihong Tian ^1,2,^*

The authors would like to apologize for any inconvenience caused to the readers by these changes.
